# Exogenous Proline Improves Salt Tolerance of Alfalfa through Modulation of Antioxidant Capacity, Ion Homeostasis, and Proline Metabolism

**DOI:** 10.3390/plants11212994

**Published:** 2022-11-07

**Authors:** Shuaiqi Guo, Xuxia Ma, Wenqi Cai, Yuan Wang, Xueqin Gao, Bingzhe Fu, Shuxia Li

**Affiliations:** 1 School of Agriculture, Ningxia University, Yinchuan 750021, China; 2 Ningxia Grassland and Animal Husbandry Engineering Technology Research Center, Yinchuan 750021, China; 3 Key Laboratory for Model Innovation in Forage Production Efficiency, Ministry of Agriculture and Rural Affairs, Yinchuan 750021, China

**Keywords:** proline, oxidative damage, K^+^/Na^+^ ratio, gene expression, OAT, salt stress

## Abstract

Alfalfa (*Medicago sativa* L.) is an important forage crop, and its productivity is severely affected by salt stress. Although proline is a compatible osmolyte that plays an important role in regulating plant abiotic stress resistance, the basic mechanism of proline requires further clarification regarding the effect of proline in mitigating the harmful effects of salinity. Here, we investigate the protective effects and regulatory mechanisms of proline on salt tolerance of alfalfa. The results show that exogenous proline obviously promotes seed germination and seedling growth of salt-stressed alfalfa. Salt stress results in stunted plant growth, while proline application alleviates this phenomenon by increasing photosynthetic capacity and antioxidant enzyme activities and decreasing cell membrane damage and reactive oxygen species (ROS) accumulation. Plants with proline treatment maintain a better K^+^/Na^+^ ratio by reducing Na^+^ accumulation and increasing K^+^ content under salt stress. Additionally, proline induces the expression of genes related to antioxidant biosynthesis (*Cu/Zn-SOD* and *APX*) and ion homeostasis (*SOS1*, *HKT1*, and *NHX1*) under salt stress conditions. Proline metabolism is mainly regulated by ornithine-δ-aminotransferase (OAT) and proline dehydrogenase (ProDH) activities and their transcription levels, with the proline-treated plants displaying an increase in proline content under salt stress. In addition, OAT activity in the ornithine (Orn) pathway rather than Δ^1^-pyrroline-5-carboxylate synthetase (P5CS) activity in the glutamate (Glu) pathway is strongly increased under salt stress, made evident by the sharp increase in the expression level of the *OAT* gene compared to *P5CS1* and *P5CS2*. Our study provides new insight into how exogenous proline improves salt tolerance in plants and that it might be used as a significant practical strategy for cultivating salt-tolerant alfalfa.

## 1. Introduction

Alfalfa (*Medicago sativa* L.) is an important and widely cultivated perennial legume forage crop around the world. Due to its high protein content, rich nutritional value, and excellent palatability for livestock, alfalfa is known as the ‘King of Forages’. The planting area of alfalfa is approximately 32.2 Mha around the world, and alfalfa has become the most important and widely used forage grass in integrated farming systems in China [1]. Alfalfa is suitable for growing in neutral or moderate saline–alkaline soil, while high salinity constrains its growth, development, and yield [2]. Compared with conventional breeding or genetic engineering methods, priming using exogenous regulators serves as an alternative method to rapidly and economically improve plant tolerance to abiotic stresses [3], which is important for the production of high-quality alfalfa on saline alkali land.

Salt stress is a major environmental factor that drastically affects plant growth, development, and productivity. It is estimated that more than 6% of the total land area and 30% of the irrigated land around the world are affected by salinity [4]. Therefore, it is necessary to improve the salt tolerance of plants to increase agricultural yields to meet the needs of the growing population and to maintain environmental sustainability. To this end, it is crucial to understand the mechanism of salt stress and the related amelioration strategies. 

Salt stress inhibits water uptake by plants, generating hyperosmotic stress and ionic toxicity because of high concentrations of toxic Na^+^ and Cl^−^ [4]. To overcome these constraints, plants have developed a variety of delicate strategies to combat unfavorable environments. The adaptive mechanisms of salt tolerance in plants mainly include (a) promoting osmotic adjustments to tolerate osmotic stress by accumulating compatible solutes in cytoplasm and vacuoles, (b) facilitating Na^+^ compartmentalization in vacuoles by NHX activity or cell extrusion by HKT and SOS1 activity, and (c) improving tissue tolerance by exporting toxic ions to vacuoles or old leaves [5,6,7]. Compatible solutes, including proline, amines, betaines, organic acids, sugars, and polyols, are low-molecular-weight osmolytes that accumulate to adjust the hypertonic stress caused by high salinity [8,9]. These solutes mainly resist the ravages of salt stress to plant cells by protecting the subcellular structure, maintaining the osmotic balance, and scavenging reactive oxygen species (ROS) [10].

A number of studies have shown that that proline plays a beneficial role in response to various environmental stresses, especially salt and water stress [10,11,12]. It mitigates the effects of ROS by initiating effective detoxification enzymatic mechanisms (SOD, APX, CAT, etc.) through osmotic adjustments and protecting cell membrane integrity [13,14,15]. Proline also serves as a major source of energy and nitrogen for plant growth under stress conditions. Not only that, but proline acts as a plant growth regulator by participating in metabolism and activating various signaling processes [13,16].

It is known that plants tend to enhance their proline levels with the continuous increase in salinity stress, which is largely dependent on the tight regulation of proline metabolism enzymes. Proline biosynthesis in plants is achieved by either the glutamate (Glu) pathway, which is mainly catalyzed by Δ^1^-pyrroline-5-carboxylate synthetase (P5CS), or by the ornithine (Orn) pathway, which is controlled by ornithine-δ-aminotransferase (OAT). Δ^1^-pyrroline-5-carboxylate reductase (P5CR), a common enzyme in both pathways, reduces pyrroline-5-carboxylate (P5C) to proline [17]. Most reports have suggested that the P5CS route accounts for major proline accumulation during stress, in which the P5CS enzyme is a rate-limiting step that is allosterically regulated by proline [18,19]. Plants typically contain two isoforms, P5CS1 and P5CS2, which mainly function in the cytosol, and P5CS1 may be transferred to chloroplasts under abiotic stress [20]. In addition, several studies have shown a pivotal role of OAT during stress conditions by modulating proline metabolism to regulate the redox homeostasis of plant cells [21].

Proline catabolism takes place in mitochondria via proline dehydrogenase (ProDH)-generating P5C, which is subsequently converted by P5C dehydrogenase (P5CDH) to Glu [18,22]. ProDH is suppressed or induced by the fluctuations in the proline concentration in plants [23,24]. Numerous studies have shown that regulating intracellular proline levels via the exogenous application of proline or overexpressing or suppressing a number of synthesis and catabolism pathway genes could mitigate the adverse effects of salt stress in plants [19,25,26]. However, there is a lack of knowledge about the mechanisms by which proline regulates the biochemical and molecular reprogramming of plants under salt stress, especially in forage plants. Based on existing reports, we speculate that the exogenous application of proline may be an effective way to improve the salt tolerance of alfalfa. In this study, we investigated the effects of exogenous proline on seed germination and the seedling growth of alfalfa under salt stress. The results showed that exogenous proline alleviated salt stress by regulating the antioxidant defense system, ion homeostasis, and proline metabolism in alfalfa.

## 2. Results

### 2.1. Exogenous Proline Promotes Alfalfa Seed Germination and Seedling Growth

**Figure 1:**
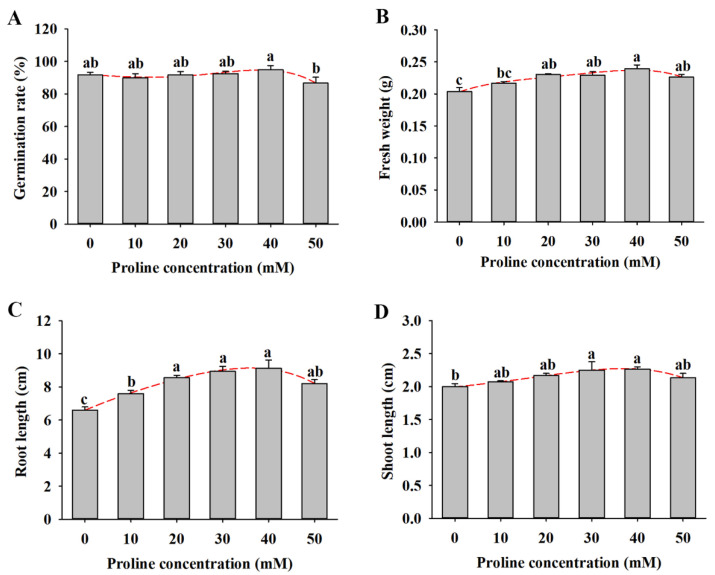
Seed germination and seedling growth of alfalfa under different concentrations of proline (0, 10, 20, 30, 40, and 50 mM) for 14 d. (**A**) Germination rate. (**B**) Fresh weight. (**C**) Root length. (**D**) Shoot length. Data are means ± SE (*n* = 40), and different letters indicate significant differences between groups (*p* < 0.05).

As shown in Figure 1A, there was no significant difference in the seed germination rate under the 0–30 mM proline treatment, which varied from 90% to 92.5%. Compared with the control, the seed germination rate reached 95% under the 40 mM proline treatment, while the rate decreased to 86.7% under the 50 mM proline treatment (Figure 1A). The fresh weight, root length, and shoot length of alfalfa seedlings with the 10–50 mM proline treatment were higher than those of untreated seedlings, and the treatment with 40 mM proline had the best performance (Figure 1B–D). 

### 2.2. Exogenous Proline Alleviates Salt Stress Inhibition on Alfalfa Seed Germination and Seedling Growth

**Figure 2:**
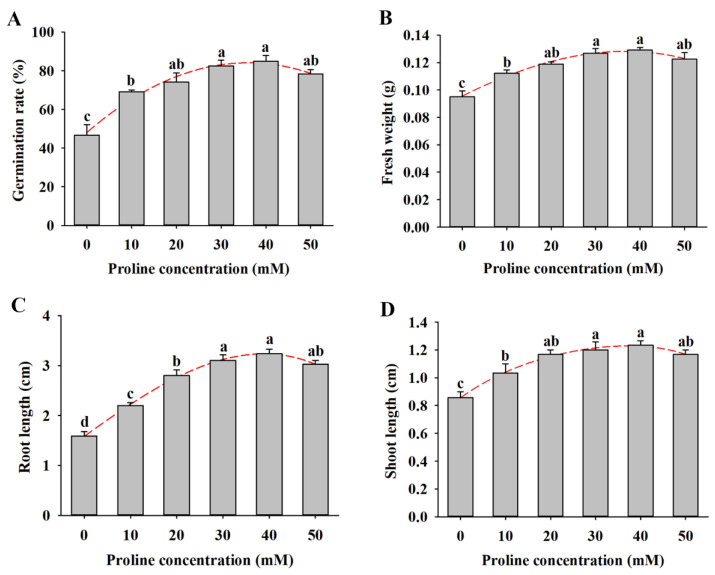
Effects of different concentrations of proline (0, 10, 20, 30, 40, and 50 mM) treatment on seed germination and seedling growth of alfalfa under salt stress for 14 d. (**A**) Germination rate. (**B**) Fresh weight. (**C**) Root length. (**D**) Shoot length. Data are means ± SE (*n* = 40), and different letters indicate significant differences between groups (*p* < 0.05).

To examine the role of proline on alfalfa seed germination and seedling growth under salt stress, the seeds were treated with different concentrations of proline (10–50 mM) under 150 mM NaCl for 14 d. Under salt stress conditions, various concentrations of proline promoted seed germination to different degrees (Figure 2A). The germination rate of alfalfa seeds under 150 mM NaCl was 46.7%, while the rates varied from 69.2% to 85% with the 10–40 mM proline pretreatments, and 50 mM proline decreased the rate to 78.3%. Proline treatment increased the fresh weight by 18.1–35.9% compared with NaCl treatment (Figure 2B). In addition, the root and shoot length of proline-treated seedlings were significantly higher than those of nontreated plants under the salt treatment, and 40 mM proline was more effective (Figure 2C,D). 

### 2.3. Exogenous Proline Improves Salt Tolerance of Alfalfa Plants

**Figure 3:**
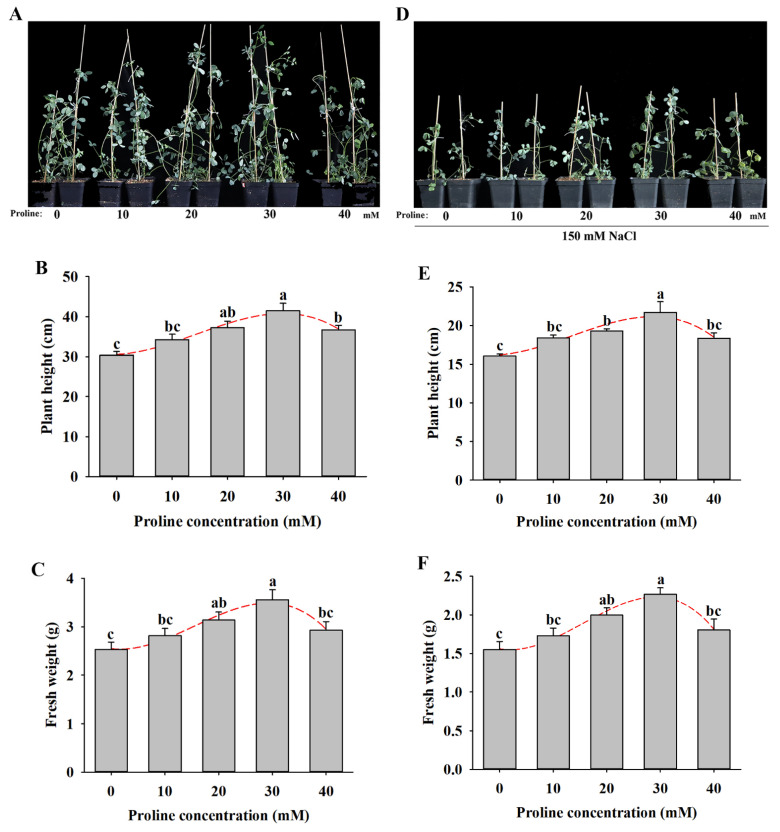
Effects of different concentrations of proline (0, 10, 20, 30, and 40 mM) treatment on soil-grown alfalfa plants under normal or salt stress conditions. (**A**–**C**) Phenotype of a representative individual from each treatment (**A**), plant height (**B**), and fresh weight (**C**) of alfalfa plants under normal conditions. (**D**–**F**) Phenotype of a representative individual from each treatment (**D**), plant height (**E**), and fresh weight (**F**) of alfalfa plants under salt stress conditions. Data are means ± SE (*n* = 3), and different letters indicate significant differences between groups (*p* < 0.05).

To investigate proline application in response to stress induced by salt, different concentrations of proline (10–40 mM) were applied to 20-day-old alfalfa plants, and the phenotypic traits were observed. Compared with the control plants, proline treatment promoted the growth of alfalfa seedings by elevating the plant height and fresh weight by 13–37% and by 11–41%, respectively (Figure 3A–C). On the contrary, salt stress inhibited the growth of plants and reduced the plant height and fresh weight of alfalfa seedings, while proline application dramatically increased both of them by 15–35% and 12–46%, respectively (Figure 3D–F). These growth indices showed that 30 mM proline was the most effective dose for further study.

### 2.4. Exogenous Proline Alleviates Oxidative Damage and Increases Antioxidant Enzyme Activities under Salt Stress

The phenotypes of proline alleviating plant growth under salt stress prompted us to analyze the physiological changes in alfalfa. Proline treatment had no significant effect on cell membrane damage, H_2_O_2_ accumulation, and antioxidant enzyme activities without salt stress (Table 1). Salt stress significantly increased the electrolyte leakage and MDA content by 2.6-fold and 4.1-fold, respectively. However, proline treatment remarkably decreased the electrolyte leakage and MDA content compared to salt-treated plants (Table 1). H_2_O_2_ accumulation was significantly increased under salt stress, but the increase was alleviated by proline treatment (Table 1). POD, SOD, and CAT are common antioxidant enzymes that scavenge excess ROS. Salt stress increased the activities of POD, SOD, and CAT, while proline treatment further elevated the activities of these enzymes compared to salt treatment alone (Table 1). 

### 2.5. Exogenous Proline Increases Photosynthetic Capacity, Soluble Sugar Contents, and K^+^/Na^+^ Ratio under Salt Stress

The maximum photochemical efficiency Fv/Fm and total chlorophyll content in alfalfa leaves was significantly decreased under salinity conditions, but the decrease was alleviated by proline treatment (Table 2). In the absence of salt stress, there was no significant difference in the soluble sugar content between control and proline-treated plants. However, the soluble sugar content was strongly increased under salt stress, and proline treatment decreased its content slightly (Table 2). 

Salt stress causes cellular excessive Na^+^ and the loss of K^+^, which leads to ion toxicity in plants. As shown in Table 2, proline had no significant effect on the Na^+^ and K^+^ contents in the absence of salt treatment. Under salt stress conditions, the Na^+^ content was significantly elevated, but it was lower in proline-treated plants than in salt-treated plants. In addition, proline-treated plants maintained higher K^+^ content in leaves under salt stress. As a consequence, the K^+^/Na^+^ ratio of SP-treated plants was significantly higher than that of S-treated plants (Table 2). 

### 2.6. Exogenous Proline Affects Proline Metabolism under Salt Stress

As expected, the proline level in plants treated with proline was considerably higher than that of the control. Salt stress resulted in a drastic increase in proline content, in which the proline content of plants treated with S and SP was 2.1- and 3.7-fold higher than that of the control (Table 3). As shown in Table 3, P5CS activity was slightly increased by salinity, but no significant difference was observed between the S-treatment and SP-treatment plants. Compared to the control plants, OAT activity was strongly increased under salt stress, whereas no modulation was observed in the proline-treatment plants. Moreover, ProDH activity was obviously decreased after salt treatment, while proline treatment resulted in significantly higher activity in the plants under normal or stress conditions (Table 3). 

### 2.7. Exogenous Proline Induces the Expression of Proline Metabolism Genes

**Figure 4:**
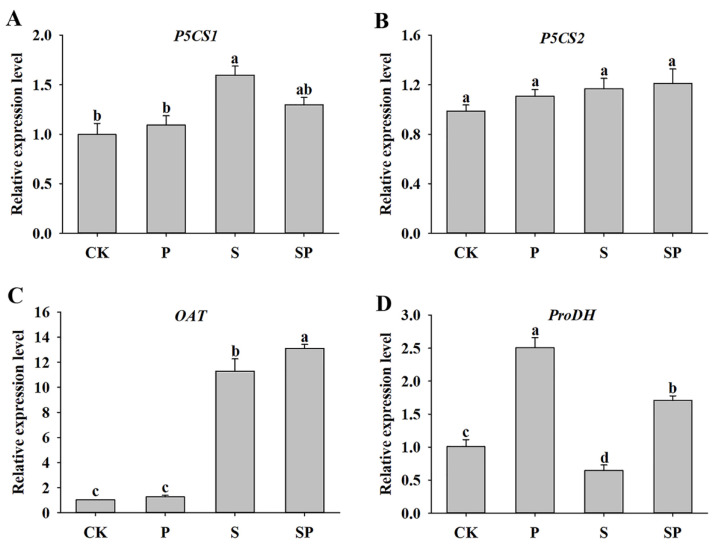
Effects of proline (30 mM) treatment on the expression levels of *P5CS1* (**A**), *P5CS2* (**B**), *OAT* (**C**), and *ProDH* (**D**) of alfalfa under salt stress. Data are means ± SE (*n* = 3), and different letters indicate significant differences between groups (*p* < 0.05).

The changes in the activity of proline metabolism enzymes under proline treatment and salt stress reminded us to analyze the relative expression levels of the proline metabolism genes. In the absence of salt treatment, exogenous proline did not significantly alter the expression level of *P5CS1* compared with the control plants (Figure 4A). However, the transcript level of the *P5CS1* gene was increased significantly by salinity, but proline application slightly inhibited *P5CS1* expression under salt stress (Figure 4A). Interestingly, *P5CS2*’s transcript levels were similar under all treatments (Figure 4B). 

Salt stress resulted in a remarkable 11-fold increase in the *OAT* expression level compared with the control. Additionally, proline treatment promoted the expression of *OAT* under salt stress conditions (Figure 4C). For *ProDH*, salt stress significantly decreased the transcript abundance, whereas proline treatment elevated its expression under normal or stress conditions (Figure 4D). 

### 2.8. Exogenous Proline Induces the Expression of Genes Related to Antioxidant Biosynthesis and Ion Homeostasis

**Figure 5:**
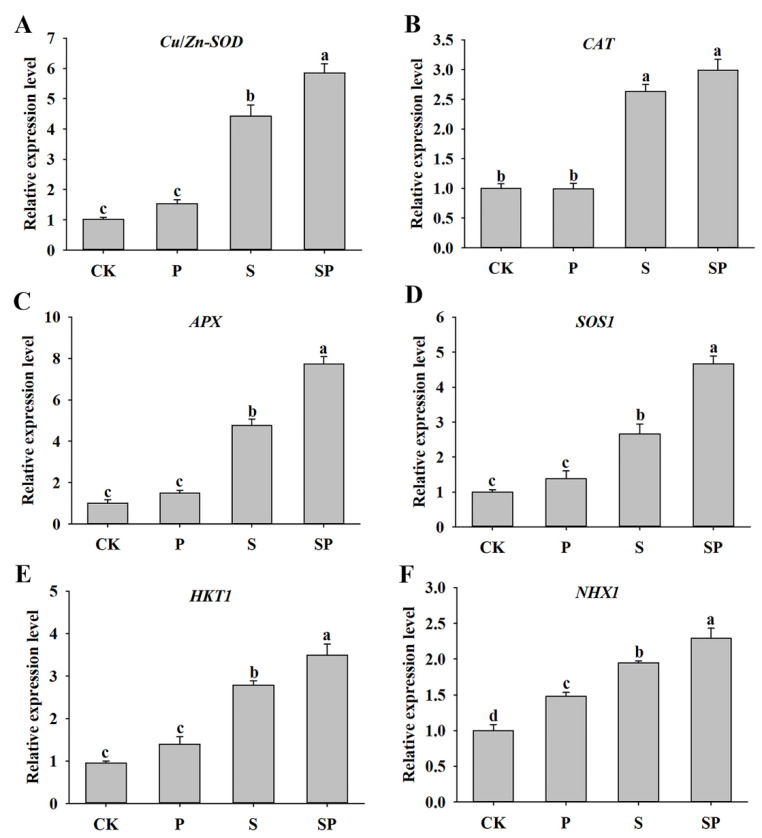
Effects of proline (30 mM) treatment on the expression levels of *Cu/Zn-SOD* (**A**), *CAT* (**B**), *APX* (**C**), *SOS1* (**D**), *HKT1* (**E**), and *NHX1* (**F**) of alfalfa under salt stress. Data are means ± SE (*n* = 3), and different letters indicate significant differences between groups (*p* < 0.05).

To further understand the role of proline in alfalfa plants’ responses to salt stress, the relative expression level of several genes related to antioxidant biosynthesis and ion homeostasis were detected. There was no significant difference in the *Cu/Zn-SOD*, *CAT,* and *APX* expression levels between the proline treatment and control plants under normal conditions. The transcripts of *Cu/Zn-SOD*, *CAT,* and *APX* were increased under the salt stress treatment, and proline treatment further upregulated *Cu/Zn-SOD* and *APX* expression levels (Figure 5A–C). The transcript levels *SOS1*, *HKT1*, and *NHX1* showed an obvious increase caused by salinity, and their expression levels in SP-treated plants were higher than those in the S-treated plants. In addition, the *NHX1* gene had higher expression in proline-treated plants than in the control plants in the absence of salt stress (Figure 5D–F). 

## 3. Discussion

In the present study, we demonstrated that exogenous proline had a protective function against salinity effects through physiochemical and molecular regulation in alfalfa. Our data showed that exogenous proline promoted seed germination and seedling growth in salt-stressed alfalfa. The improved salt tolerance of alfalfa seedlings by exogenous proline was mainly attributed to the enhanced antioxidant defense system, the maintenance of K/Na ion homeostasis, and the regulation of proline metabolism, with the last process mainly requiring the precise regulation of specific gene members (*OAT* and *ProDH*) and enzymes (OAT and ProDH) to maintain optimal proline levels against salt stress. 

Salinity is a widespread environmental stress factor that severely impedes plant growth and development from seed germination to harvest. Seed germination is one of the most critical stages of physiological metabolism in the plant life cycle because it is particularly sensitive to the external environment [27]. Osmotic stress and the high ion accumulation (Na^+^ and Cl^−^) caused by salinity limit seed water absorption and produce ion toxicity [28]. Proline is the most common osmotic adjustment substance and plays an important role in regulating plant abiotic stress resistance, including salinity [29,30]. Only a few studies have shown that exogenous proline alleviates the inhibition of salinity during seed germination in plants such as sorghum, rice, and hyacinth bean [10,31,32]. In the current study, we found that exogenous proline also promoted seed germination and seedling growth in salt-stressed alfalfa. In addition, proline demonstrated a dose-dependent function on seed germination and seedling growth under salt conditions, and 40 mM was the optimum concentration in alfalfa (Figure 2). Similarly, a 1 mM proline application was more beneficial in alleviating the negative effect of salinity in rice [33]. Proline soaking alleviated the inhibition of α-amylase activity via low temperatures and promoted the germination of maize seeds [30]. Proline treatment increased the fresh weight of alfalfa seedlings under salt stress, suggesting that it may inhibit the osmotic stress induced by salt stress. Thus, we demonstrate that proline ameliorates the negative effects of salinity on seed germination and seedling growth in alfalfa.

Salinity alters physiological and biochemical responses in plants, which reflects the ability of plants to resist salt stress to some extent. The excessive reactive oxygen species (ROS) accumulation and lipid peroxidation in plants under salt stress usually leads to cell membrane damage and oxidative stress [33]. Electrolyte leakage and MDA are important indicators of cell membrane peroxidation under salt stress [34]. Proline has been considered as a molecular chaperone because of its capacity to scavenge ROS, to stabilize DNA, membranes, and protein complexes, and to provide intracellular redox potential [15,35]. Herein, we found that the content of H_2_O_2_ in the proline-treated plants was slightly lower than that in the control, although there no significant differences were observed (Table 1), which might be attributed to the capacity of proline as a chaperone to scavenge ROS, something that was also confirmed by Sorkheh et al. [36]. Moreover, electrolyte leakage and the MDA and H_2_O_2_ contents were decreased with proline treatment under salt stress (Table 1), implying that proline application alleviated the oxidative damage caused by ROS. Plants activate antioxidant enzymes (such as POD, SOD, CAT, etc.) to defend against ROS damage under stressful conditions [33]. The notable high levels of POD and SOD activity in the proline-treated plants under salt stress indicated that proline enhanced the capacity of ROS scavenging (Table 1). The results corresponded to the lower H_2_O_2_ content in proline-treated plants under salt stress conditions. Furthermore, the transcripts of the *Cu/Zn-SOD* and *APX* genes were upregulated significantly in the plants that received proline treatment under salt stress (Figure 5A,C), which contributed to enhancing the antioxidant capacity to scavenge ROS. These results are similar to the data produced by Wani et al. [37], where exogenous proline improved the salt tolerance of *B. juncea* cultivars by decreasing electrolyte leakage and enhancing CAT, SOD, and POX activity, indicating that proline and antioxidant enzymes have interacting networks. These data suggest that exogenous proline alleviates the oxidative damage caused by ROS through enhancing the activity of antioxidant enzymes under salt stress. 

High salt concentrations cause the excessive accumulation of cytosolic Na^+^ and the loss of K^+^ in plants, leading to ionic imbalance and dysfunction in plants [38]. Indeed, maintaining ion homeostasis, and an optimal K^+^/Na^+^ ratio in particular, is one of the critical adaptive strategies to cope with salt stress [39]. Herein, we observed that proline-treated plants had lower concentrations of Na^+^ and higher K^+^ contents, resulting in a higher K^+^/Na^+^ ratio under salt stress (Table 2). It is well-documented that removing Na^+^ from cytoplasm and compartmentalizing it in the vacuoles are important strategies to maintain a low Na^+^ concentration [40,41]. In this study, transcript abundances of *SOS1*, *HKT1*, and *NHX1* were significantly increased in the proline treatment plants under salt stress (Figure 5D–F), demonstrating that proline might contribute to reducing Na^+^ in the cytosol by cell extrusion and vacuole compartmentalization, thus protecting the plants from Na^+^ toxicity. These results match perfectly with Na^+^ accumulation and support better salt tolerance in the plants treated with proline. Similar results have been reported in maize and aloe vera plants [12,42]. Additionally, Ahmed et al. [43] reported that exogenous proline largely reduced Na^+^ accumulation in leaves and roots because of its interference or dilution during osmotic adjustment. The modulation of exogenous proline on photosynthetic activity under salt stress is closely related to favorable ionic homeostasis [10], and the effect was manifested by the higher preservation of the maximum photochemical efficiency and chlorophyll content in this study (Table 2). Thus, the alleviation of salt damage by exogenous proline is associated with Na^+^ and K^+^ homeostasis.

Proline accumulation is particularly associated with adaptation to salt stress in many plant species [19,25,26]. It has been suggested that proline is involved in the osmotic potential of leaves, and consequently, in osmotic adjustment, plants benefit from a greater proline content under salt stress [44]. Proline accumulated during a stress episode is degraded to provide a supply of energy to drive growth once the stress is relieved. Proline homeostasis is critical for active cell division and sustainable growth under long-term stress [19,45]. In this study, proline treatment increased the proline content of alfalfa leaves under normal or salt stress conditions (Table 3). Studies have shown that when the proline produced by plants is not enough to eliminate the damage caused by stress, it can be alleviated by the exogenous application of proline [19,46]. This may be the reason why proline application increased the proline content in alfalfa leaves under salt stress. Proline levels in response to abiotic stress are mainly controlled by key synthesis and degradation enzymes and their transcriptional regulation [10,47]. Studies have showed that the Glu pathway rather than the Orn pathway is essential for proline biosynthesis during stress regulation in most species [10,48,49]. However, in this study, the significant increase in OAT activity and *OAT* transcription induced by salinity indicated that the Orn pathway was predominant in alfalfa plants (Table 3, Figure 4C). In addition, the Glu pathway was also involved in proline biosynthesis, made evident by elevated P5CS activity and *P5CS1* expression after NaCl imposition (Table 3, Figure 4A). Our results are consistent with the finding that the OAT pathway plays a central role in legumes, as reported by Abdelgawad et al. [17].

The function of proline is dose dependent, and a high concentration could cause a toxic effect in plants [10,50]. ProDH activity and *ProDH* gene expression were decreased under salt stress; however, proline treatment greatly increased the activity of ProDH and the transcription of the *ProDH* gene (Table 3, Figure 4D), implying that this response may protect plants against excessive proline toxicity [12,51]. Additionally, the decreases in P5CS activity and *P5CS1* expression under the combined salt stress and proline treatment were also the embodiment of proline detoxification (Table 3, Figure 4A). Nevertheless, the modulation of ProDH and P5CS was not enough to reduce the proline content of alfalfa leaves. Similarly, exogenous proline application decreased P5CS activity and increased ProDH activity under salt stress in maize [12]. Therefore, the higher proline levels in salt- and proline-treated alfalfa were maintained by the modulation of biosynthetic enzymes (OAT and P5CS1) and the catabolic enzyme (ProDH) and their transcription. Overall, our research suggests that the exogenous application of proline has a positive role in overcoming the negative effects of salt stress on the seed germination, seedling growth, and physiological parameters of alfalfa plants. 

## 4. Materials and Methods

### 4.1. Germination Test and Measurement of Growth Parameter

Alfalfa (*Medicago sativa* L.) variety Zhongmu No. 1 was used as the material in this experiment. Zhongmu No. 1 was obtained by hybridizing several kinds of alfalfa widely grown in northern China. Zhongmu No. 1 is a heterozygous autotetraploid, which has the characteristics of salt tolerance, drought resistance, and rapid growth. Alfalfa seeds were surface-sterilized with 75% (*v*/*v*) ethanol for 30 s and with 5% sodium hypochlorite solution for 10 min, after which they were thoroughly rinsed with sterile water 4–5 times. Forty sterilized seeds were placed on Petri dishes with filter paper moistened with 10 mL of the treatment solutions and were germinated in a growth incubator (PGX-1000B, Ningbo Saifu Experimental Instrument Co., Ltd., Ningbo, China) at 60% humidity and a 16/8 h light/dark photoperiod at 25 °C. A total of 12 treatments were set up in the germination experiment, including distilled water (control); proline: 10, 20, 30, 40, and 50 mM (P1, P2, P3, P4, and P5); 150 mM NaCl (salinity); and combined treatments: SP1, SP2, SP3, SP4, and SP5. The treatment solution was replaced every two days, and three replicates were performed. The germination rates, fresh weight, and root and shoot lengths of the seedlings were measured on the 14th day posttreatment.

### 4.2. Proline Application and Salt Treatment of Alfalfa Plants

Alfalfa seeds were germinated on filter paper and then transplanted into plastic pots that were 7 cm in diameter and 8.5 cm in depth with vermiculite in a growth room with a 16/8 h light/dark photoperiod at 25 °C. The seedlings were watered with modified Hoagland nutrient solution. The main components of the modified Hoagland nutrient solution include 4 mM Ca(NO_3_)_2_, 6 mM KNO_3_, 0.5 mM NaH_2_PO_4_, 2 mM MgSO_4_, 0.1 mM NH_4_NO_3_, trace elements, and iron salts. Twenty days after pre-cultivation, when the seedlings were at the five-leaf stage, the treatments were started. Four treatments were performed on alfalfa seedlings with uniform growth: (1) control (CK), sprayed with 10 mL of deionized water; (2) proline (P), sprayed with 10 mL of proline solution; (3) salt stress (S), sprayed with 10 mL of deionized water and salt-stressed; and (4) salt stress with proline (SP), sprayed with 10 mL of proline solution and salt-stressed. The seedlings were sprayed with deionized water or proline solution twice a day (at 8:00 a.m. and 6:00 p.m.) for 7 days and then treated with 150 mM NaCl every 3 days for 14 days. To select the optimal concentration of proline, four different concentrations (10, 20, 30, and 40 mM) were applied in the preliminary experiment. According to the growth phenotype of alfalfa, the representative individuals from each group were photographed by a camera on the 14th day of salt treatment, and then the plant height and fresh weight were measured (the branching stage of vegetative growth). The alfalfa leaves were harvested and instantly frozen in liquid nitrogen and stored at −80 °C to determine further physiological parameters and gene expression analysis. 

### 4.3. Damage Assessment and H_2_O_2_ Determination

Electrolyte leakage was determined according to the method of Dahro et al. [52]. The content of malondialdehyde (MDA) was determined using the thiobarbituric acid (TBA) method as described by Puckette et al. [53]. The H_2_O_2_ concentration was quantified following the method described by Jiang and Zhang [54].

### 4.4. Determination of Antioxidant Enzyme Activities

The antioxidant enzyme activities were determined by spectrophotometry. The superoxide dismutase (SOD) activity was measured using the nitroblue tetrazolium (NBT) method according to Dhindsa et al. [55]. The peroxidase (POD) activity was determined using the guaiacol method according to Polle et al. [56]. Catalase (CAT) activity was analyzed by monitoring the consumption of H_2_O_2_ according to Jiang and Zhang [54].

### 4.5. Determination of Photosynthetic Capacity and Soluble Sugar Contents

The maximum photochemical efficiency (Fv/Fm) was measured using the IMAGING-PAM Chlorophyll Fluorometer (Walz, Germany) following the manufacturer’s instructions. Leaf total chlorophyll was extracted with 80% acetone and analyzed according to Liu et al. [57], with minor modifications. Briefly, nearly 0.1 g of fresh leaf power was suspended in 5 ml of 80% acetone and extracted for 20 min at room temperature in the dark. The crude extraction was centrifuged at 5000× *g* for 10 min, and the absorbance of the supernatant was measured at 663, 645, and 480 nm. The soluble sugar content was extracted with 80% ethanol and measured using anthrone reagent, as described by Dreywood [58]. 

### 4.6. Determination of Na^+^ and K^+^ Contents

The Na^+^ and K^+^ content was measured according to Li et al. [41]. Leaf samples were dried at 80 °C for 2 days, and approximately 50 mg of dry powder samples were dissolved with acetic acid solution for 1 h. The acid-digested products were centrifuged at 15,000 rpm for 15 min, and the supernatants were analyzed by using a flame spectrophotometer (FP6450, Shanghai, China).

### 4.7. Determination of Proline Content and Metabolism Enzyme Activities

Free proline was extracted with sulfosalicylic acid, and the content was determined using the ninhydrin method according to Bates et al. [59]. Proline metabolism enzyme activity was measured spectrometrically according to de Freitas et al. [10]. P5CS activity was measured by monitoring the increase in absorbance at 340 nm caused by NADPH oxidation. One unit of P5CS activity was defined as an increase of 0.001 absorbance units per minute. OAT activity was measured using a standard curve designed with known P5C concentrations. One unit of OAT activity was defined as the amount of enzyme required to reduce 1.0 µmol of P5C per hour. ProDH activity was measured by monitoring the decrease in absorbance at 600 nm that was due to the reduction in dichloroindophenol (DCIP) by proline oxidation. One unit of ProDH activity was defined as the amount of enzyme catalyzing the reduction of 1.0 mol of DCIP per minute. The results were expressed as U mg^−1^ protein. The protein concentrations in the plant crude extracts were determined according to Bradford et al. [60].

### 4.8. Quantitative Real-Time PCR (qRT-PCR) Analysis

Total RNA was isolated from alfalfa leaf tissues using the MiniBEST Plant RNA Extraction Kit (Takara). The cDNA was synthesized using the HiScript II 1st Strand cDNA Synthesis Kit (+gDNA wiper) (Vazyme). qRT-PCR was performed on Bio-Rad CFX 96 (Bio-Rad, Inc., CA, USA) using the ChamQ SYBR qPCR Master Mix (Vazyme). The alfalfa β-actin gene (MsActin) was used as a reference gene, and the relative expression levels of genes were calculated by the 2^−ΔΔCT^ method [61]. Three independent biological replicates and three replicate reactions were performed for each sample. The gene-specific primers used in this study are listed in Table S1.

### 4.9. Statistical Analysis

The data for the experiment were analyzed using SPSS Statistical 20.0 software, and significant differences were calculated based on one-way ANOVA followed by Duncan’s tests. Differences between individual means were considered significant at *p* < 0.05. Figures were created using SigmaPlot software (Version 12.5).

## 5. Conclusions

In summary, we identified the protective effects of proline on alfalfa seed germination and seedling growth against salt stress. Proline played a dose-dependent function in alleviating salt stress damage, and 40 mM and 30 mM were the optimum concentrations for alfalfa seed germination and plant growth, respectively. Exogenous proline application alleviated the salt damage of alfalfa by modulating antioxidant enzyme activities and ion homeostasis. Moreover, exogenous proline could regulate endogenous proline metabolism, mainly by regulating OAT and ProDH activities and their transcription levels under salt stress, and the Orn pathway was predominant in alfalfa plants. The current study provides insights into the biochemical and molecular mechanisms underlying exogenous proline regulation in response to salt stress and also provides an important strategy for cultivating salt-tolerant alfalfa.

## Figures and Tables

**Table 1:** Effects of proline (30 mM) treatment on electrolyte leakage (EL), MDA content, H_2_O_2_ content, POD activity, SOD activity, and CAT activity of alfalfa under salt stress.

Treatments	EL (%)	MDA (μmol g^−1^ FW)	H_2_O_2_ (μmol g^−1^ FW)	POD (U g^−1^ FW)	SOD (U g^−1^ FW)	CAT (U g^−1^ FW)
CK	24.85 ± 0.76 c	0.18 ± 0.01 c	2.72 ± 0.20 c	29767.64 ± 1668.01 c	246.66 ± 11.20 c	83.02 ± 4.79 b
P	26.45 ± 0.36 c	0.18 ± 0.01 c	2.49 ± 0.18 c	29618.03 ± 477.89 c	250.58 ± 15.81 c	84.58 ± 8.83 b
S	65.31 ± 1.97 a	0.73 ± 0.03 a	4.10 ± 0.14 a	38292.24 ± 966.53 b	369.54 ± 10.85 b	102.36 ± 2.05 ab
SP	57.58 ± 1.13 b	0.49 ± 0.05 b	3.48 ± 0.20 b	45531.07 ± 2575.21 a	415.32 ± 15.40 a	122.03 ± 8.82 a

Data are means ± SE (*n* = 3), and different letters indicate significant differences between treatments (*p* < 0.05).

**Table 2:** Effects of proline (30 mM) treatment on the Fv/Fm, chlorophyll content, soluble sugar content, Na^+^ content, K^+^ content, and K^+^/Na^+^ ratio of alfalfa under salt stress.

Treatments	Fv/Fm	Chlorophyll (mg g^−1^ FW)	Soluble Sugar (μg g^−1^)	Na^+^ (mg g^−1^ DW)	K^+^ (mg g^−1^ DW)	K^+^/Na^+^
CK	0.82 ± 0.01 a	5.01 ± 0.12 a	436.57 ± 43.15 bc	15.96 ± 1.20 c	47.76 ± 3.19 a	3.00 ± 0.09 a
P	0.84 ± 0.01 a	5.08 ± 0.12 a	396.82 ± 21.63 c	14.82 ± 0.52 c	48.55 ± 1.75 a	3.28 ± 0.16 a
S	0.66 ± 0.02 c	3.76 ± 0.14 c	576.50 ± 18.75 a	51.47 ± 1.82 a	27.00 ± 1.45 c	0.53 ± 0.04 c
SP	0.74 ± 0.01 b	4.51 ± 0.12 b	527.71 ± 39.56 ab	40.39 ± 1.85 b	35.06 ± 1.19 b	0.87 ± 0.06 b

Data are means ± SE (*n* = 3), and different letters indicate significant differences between groups (*p* < 0.05).

**Table 3:** Effects of proline (30 mM) treatment on proline content, P5CS activity, OAT activity, and ProDH activity of alfalfa under salt stress.

Treatments	Proline Content (μg g^−1^ FW)	P5CS (U mg^−1^ Protein)	OAT (U mg^−1^ Protein)	ProDH (U mg^−1^ Protein)
CK	195.87 ± 24.68 c	58.08 ± 2.94 b	11.45 ± 0.63 b	48.71 ± 2.60 b
P	333.90 ± 22.84 b	62.36 ± 3.19 ab	10.74 ± 0.91 b	68.85 ± 1.98 a
S	410.15 ± 15.22 b	68.89 ± 1.09 a	19.51 ± 0.46 a	35.14 ± 3.08 c
SP	727.81 ± 39.31 a	65.14 ± 3.92 ab	18.64 ± 1.58 a	53.54 ± 2.76 b

Data are means ± SE (*n* = 3), and different letters indicate significant differences between treatments (*p* < 0.05).

## Data Availability

Not applicable.

## Supplementary Materials

The following supporting information can be downloaded at: https://www.mdpi.com/article/10.3390/plants11212994/s1, Table S1: List of primers used in qRT-PCR.

## References

[1] Cen H.F., Wang T.T., Liu H.Y., Tian D.Y., Zhang Y.W. (2020). Melatonin application improves salt tolerance of alfalfa (*Medicago sativa* L.) by enhancing antioxidant capacity. Plants.

[2] Singer S.D., Hannoufa A., Acharya S. (2018). Molecular improvement of alfalfa for enhanced productivity and adaptability in a changing environment. Plant Cell Environ..

[3] Fu J.J., Zhang S.T., Jiang H.N., Zhang X.F., Gao H., Yang P.Z., Hu T.M. (2022). Melatonin-induced cold and drought tolerance is regulated by brassinosteroids and hydrogen peroxide signaling in perennial ryegrass. Environ. Exp. Bot..

[4] Fahad S., Hussain S., Matloob A., Khan F.A., Khaliq A., Saud S., Hassan S., Shan D., Khan F., Ullah N. (2014). Phytohormones and plant responses to salinity stress: A review. Plant Growth Reg..

[5] Munns R., Tester M. (2008). Mechanisms of salinity tolerance. Annu. Rev. Plant Biol..

[6] Miranda R.S., Mesquita R.O., Costa J.H., Alvarez-Pizarro J.C., Prisco J.T., Gomes-Filho E. (2017). Integrative control between proton pumps and SOS1 antiporters in roots is crucial for maintaining low Na^+^ accumulation and salt tolerance in ammonium-supplied *Sorghum bicolor*. Plant Cell Physiol..

[7] Zhao C.Z., Zhang H., Song C.P., Zhu J.K., Shabala S. (2020). Mechanisms of plant responses and adaptation to soil salinity. Innovation.

[8] Parihar P., Singh S., Singh R., Singh V.P., Prasad S.M. (2015). Effect of salinity stress on plants and its tolerance strategies: A review. Environ. Sci. Pollut. Res..

[9] Park H.J., Kim W.Y., Yun D.J. (2016). A new insight of salt stress signaling in plant. Mol. Cells.

[10] de Freitas P.A.F., de Carvalho H.H., Costa J.H., Miranda R.S., Saraiva K.D.C., Oliveira F.D.B., Coelho D.G., Prisco J.T., Gomes-Filho E. (2019). Salt acclimation in sorghum plants by exogenous proline: Physiological and biochemical changes and regulation of proline metabolism. Plant Cell Rep..

[11] Borgo L., Marur C.J., Vieira L.G.E. (2015). Effects of high proline accumulation on chloroplast and mitochondrial ultrastructure and on osmotic adjustment in tobacco plants. Acta Sci. Agron..

[12] de Freitas P.A.F., Miranda R.S., Marques E.C., Prisco J.T., Gomes-Filho E. (2018). Salt tolerance induced by exogenous proline in maize is related to low oxidative damage and favorable ionic homeostasis. J. Plant Growth Regul..

[13] Szabados L., Savoure A. (2010). Proline: A mulitfunctional amino acid. Trends Plant Sci..

[14] Reddy P.S., Jogeswar G., Rasineni G.K., Maheswari M., Reddy A.R., Varshney R.K., Kishor P.B.K. (2015). Proline over-accumulation alleviates salt stress and protects photosynthetic and antioxidant enzyme activities in transgenic sorghum [*Sorghum bicolor* (L.) Moench]. Plant Physiol. Biochem..

[15] Hosseinifard M., Stefaniak S., Ghorbani J.M., Soltani E., Wojtyla L., Garnczarska M. (2022). Contribution of exogenous proline to abiotic stresses tolerance in plants: A review. Int. J. Mol. Sci..

[16] Sharma S., Villamor J.G., Verslues P.E. (2011). Essential role of tissue-specific proline synthesis and catabolism in growth and redox balance at low water potential. Plant Physiol..

[17] AbdElgawad H., De Vos D., Zinta G., Domagalska M.A., Beemster G.T.S., Asard H. (2015). Grassland species differentially regulate proline concentrations under future climate conditions: An integrated biochemical and modelling approach. New Phytol..

[18] Hayat S., Hayat Q., Alyemeni M.N., Wani A.S., Pichtel J., Ahmad A. (2012). Role of proline under changing environments a review. Plant Signal Behav..

[19] Mansour M.M.F., Ali E.F. (2017). Evaluation of proline functions in saline conditions. Phytochemistry.

[20] Szekely G., Abraham E., Cseplo A., Rigo G., Zsigmond L., Csiszar J. (2008). Duplicated *P5CS* genes of Arabidopsis play distinct roles in stress regulation and developmental control of proline biosynthesis. Plant J..

[21] Yamada M., Morishita H., Urano K., Shiozaki N., Yamaguchi-Shinozaki K., Shinozaki K., Yoshiba Y. (2005). Effects of free proline accumulation in petunias under drought stress. J. Exp. Bot..

[22] Kishor P.B.K., Sreenivasulu N. (2014). Is proline accumulation per se correlated with stress tolerance or is proline homeostasis a more critical issue?. Plant Cell Environ..

[23] Kiyosue T., Yoshiba Y., Yamaguchi-Shinozaki K., Shinozaki K. (1996). A nuclear gene encoding mitochondrial proline dehydrogenase, an enzyme involved in proline metabolism, is upregulated by proline but downregulated by dehydration in Arabidopsis. Plant Cell.

[24] Kishor P.B.K., Sangam S., Amrutha R., Laxmi P.S., Naidu K.R., Rao K.R.S.S. (2005). Regulation of proline biosynthesis, degradation, uptake and transport in higher plants: Its implications in plant growth and abiotic stress tolerance. Curr. Sci..

[25] Rejeb K.B., Vos D.L., Disquet I.L., Leprince A.S., Bordenave M., Maldiney R., Jdey A., Abdelly C., Savoure A. (2015). Hydrogen peroxide produced by NADPH oxidases increases proline accumulation during salt or mannitol stress in *Arabidopsis thaliana*. New Phytol..

[26] Dai W.S., Wang M., Gong X.Q., Liu J.H. (2018). The transcription factor FcWRKY40 of *Fortunella crassifolia* functions positively in salt tolerance through modulation of ion homeostasis and proline biosynthesis by directly regulating *SOS2* and *P5CS1* homologs. New Phytol..

[27] Moukhtari A.E., Cabassa-Hourton C., Farissi M., Savouré A. (2020). How does proline treatment promote salt stress tolerance during crop plant development?. Front. Plant Sci..

[28] Farissi M., Bouizgaren A., Faghire M., Bargaz A., Ghoulam C. (2011). Agrophysiological responses of Moroccan alfalfa (*Medicago sativa* L.) populations to salt stress during germination and early seedling stages. Seed Sci. Technol..

[29] Slama S., Bouchereau A., Flowers T., Abdelly C., Savouré A. (2015). Diversity, distribution and roles of osmoprotective compounds accumulated in halophytes under abiotic stress. Ann. Bot..

[30] Zuo S.Y., Li J., Gu W.R., Wei S. (2022). Exogenous proline alleviated low temperature stress in maize embryos by optimizing seed germination, inner proline metabolism, respiratory metabolism and a hormone regulation mechanism. Agriculture.

[31] Athar H., Ashraf M., Wahid A., Jamil A. (2009). Inducing salt tolerance in canola (*Brassica napus* L.) by exogenous application of glycinebetaine and proline: Response at the initial growth stages. Pak. J. Bot..

[32] Deivanai S., Xavier R., Vinod V., Timalata K., Lim O. (2011). Role of exogenous proline in ameliorating salt stress at early stage in two rice cultivars. J. Stress Physiol. Biochem..

[33] Miller G., Suzuki N., Ciftci-Yilmaz S., Mittler R. (2010). Reactive oxygen species homeostasis and signalling during drought and salinity stresses. Plant Cell Environ..

[34] Ashrafi E., Razmjoo J., Zahedi M., Pessarakli M. (2015). Screening alfalfa for salt tolerance based on lipid peroxidation and antioxidant enzymes. Agron. J..

[35] Rejeb K.B., Abdelly C., Savouré A. (2014). How reactive oxygen species and proline face stress together. Plant Physiol. Biochem..

[36] Sorkheh K., Shiran B., Khodambashi M., Rouhi V., Mosavei S., Sofo A. (2012). Exogenous proline alleviates the effects of H_2_O_2_-induced oxidative stress in wild almond species. Russ. J. Plant Physiol..

[37] Wani A.S., Ahmad A., Hayat S., Tahir I. (2016). Is foliar spray of proline sufficient for mitigation of salt stress in *Brassica juncea* cultivars?. Environ. Sci. Pollut. Res..

[38] Zhu J.K. (2002). Salt and drought stress signal transduction in plants. Annu. Rev. Plant Biol..

[39] Yang Y., Guo Y. (2018). Elucidating the molecular mechanisms mediating plant salt-stress responses. New Phytol..

[40] Bargaz A., Zaman-Allah M., Farissi M., Lazali M., Drevon J.J., Maougal T.R., Georg C. (2015). Physiological and molecular aspects of tolerance to environmental constraints in grain and forage legumes. Int. J. Mol. Sci..

[41] Li S.X., Liu J.L., An Y.R., Cao Y.M., Liu Y.S., Zhang J., Geng J.C., Hu T.M., Yang P.Z. (2019). *MsPIP2; 2*, a novel aquaporin gene from *Medicago sativa*, confers salt tolerance in transgenic Arabidopsis. Environ. Exp. Bot..

[42] Nakhaie A., Habibi G., Vaziri A. (2022). Exogenous proline enhances salt tolerance in acclimated *Aloe vera* by modulating photosystem II efficiency and antioxidant defense. S. Afr. J. Bot..

[43] Ahmed B.C., Magdich S., Ben Rouina B., Sensoy S., Boukhris M., Ben Abdullah F. (2011). Exogenous proline effects on water relations and ions contents in leaves and roots of young olive. Amino Acids.

[44] Aboryia M.S., El-Dengawy E.-R.F.A., El-Banna M.F., El-Gobba M.H., Kasem M.M., Hegazy A.A., Hassan H.M., El-Yazied A.A., El-Gawad H.G.A., Al-Qahtani S.M. (2022). Anatomical and physiological performance of Jojoba treated with proline under salinity stress condition. Horticulturae.

[45] Tarchevsky I.A., Egorova A.M. (2022). Participation of proline in plant adaptation to stress factors and its application in agrobiotechnology (Review). Appl. Biochem. Microbiol..

[46] Mishra S., Dubey R.S. (2006). Inhibition of ribonuclease and protease activities in arsenic exposed rice seedlings: Role of proline as enzyme protectant. J. Plant Physiol..

[47] Sharma S., Verslues P.E. (2010). Mechanisms independent of abscisic acid (ABA) or proline feedback have a predominant role in transcriptional regulation of proline metabolism during low water potential and stress recovery. Plant Cell Environ..

[48] Funck D., Stadelhofer B., Koch W. (2008). Ornithine-δ-aminotransferase is essential for Arginine Catabolism but not for Proline Biosynthesis. BMC Plant Biol..

[49] Zhen W.B., Ma Q.H. (2009). Proline metabolism in response to salt stress in common reed [*Phragmites australis* (Cav.) Trin. Ex Steud]. Bot. Mar..

[50] Maggio A., Miyazaki S., Veronese P., Fujita T., Ibeas J., Damsz B., Narasimhan M.L., Hasegawa P.M., Joly R.J., Bressan R. (2002). Does proline accumulation play an active role in stress-induced growth reduction?. Plant J..

[51] Rady M., Kusvuran A., Alharby H.F., Alzahrani Y., Kusvuran S. (2019). Pretreatment with proline or an organic bio-stimulant induces salt tolerance in wheat plants by improving antioxidant redox state and enzymatic activities and reducing the oxidative stress. J. Plant Growth Regul..

[52] Dahro B., Wang F., Peng T., Liu J. (2016). *PtrA/NINV*, an alkaline/neutral invertase gene of *Poncirus trifoliata*, confers enhanced tolerance to multiple abiotic stresses by modulating ROS levels and maintaining photosynthetic efficiency. BMC Plant Biol..

[53] Puckette M.C., Weng H., Mahalingam R. (2007). Physiological and biochemical responses to acute ozone-induced oxidative stress in *Medicago truncatula*. Plant Physiol. Biochem..

[54] Jiang M.Y., Zhang J.H. (2001). Effect of abscisic acid on active oxygen species, antioxidative defence system and oxidative damage in leaves of maize seedlings. Plant Cell Physiol..

[55] Dhindsa R.S., Plumb-Dhindsa P., Thorpe T.A. (1981). Leaf senescence: Correlated with increased levels of membrane permeability and lipid peroxidation, and decreased levels of superoxide dismutase and catalase. J. Exp. Bot..

[56] Polle A., Otter T., Seifert F. (1994). Apoplastic peroxidases and lignification in needles of norway spruce (*Picea abies* L.). Plant Physiol..

[57] Liu J.H., Inoue H., Moriguchi T. (2008). Salt stress-mediated changes in free polyamine titers and expression of genes responsible for polyamine biosynthesis of apple in vitro shoots. Environ. Exp. Bot..

[58] Dreywood R. (1946). Qualitative test for carbohydrate material. Ind. Eng. Chem. Anal. Ed..

[59] Bates L.S., Waldren R.P., Teare I.D. (1973). Rapid determination of free proline for water-stress studies. Plant Soil.

[60] Bradford M.M. (1976). A rapid and sensitive method for the quantitation of microgram quantities of protein utilizing the principle of protein-dye binding. Anal. Biochem..

[61] Livak K.J., Schmittgen T.D. (2001). Analysis of relative gene expression data using real-time quantitative PCR and the 2^−ΔΔCt^ Method. Methods.

