# Supplementary material for: Exogenous Proline Improves Salt Tolerance of Alfalfa through Modulation of Antioxidant Capacity, Ion Homeostasis, and Proline Metabolism
Source: Plants (Basel). 2022 Nov 7;11(21):2994. doi: 10.3390/plants11212994 (PMC9657615; doi:10.3390/plants11212994)
Supplement: Supplementary file 1 [file plants-11-02994-s001.zip › Supplementary Table S1.pdf]

Supplementary Material

Table S1. List of primers used in qRT-PCR.

Primer Name	Primer sequences (5′-3′)
P5CS1-F	AGATTGGGCAGAGGGGGTAT
P5CS1-R	GAACTGTTACGTGCTGCGAC
P5CS2-F	TGGTCCCTCGAGGCAGTAAT
P5CS2-R	TCGTTCAGTCCACCGTTTCC
OAT-F	TGGAGTCGAGCTTAACAGCG
OAT-R	AGGAGCAGCATCTTTTGGCT
ProDH-F	TGGACGTTGGAATATGGGCTA
ProDH-R	AAGCATGCCACGTAAGCCA
Cu/Zn-SOD-F	GATGGAACCGCAAGCTTCAC
Cu/Zn-SOD-R	GCTCATGACCACCTTTCCCA
CAT-F	TTACAGCCCGATCCAACGAC
CAT-R	GCCAATGACACTTCCCATGC
APX-F	TGAAGTTCCTTTCCACCCCG
APX-R	TAGAAGTCCATGGGCCCTCA
SOS1-F	TGGAGTTGGAAAACGTCGCT
SOS1-R	ACGCAAGGCCAATTCCTACA
HKT1-F	TGCTTATGGGAACGTAGGGT
HKT1-R	CAGGCTTTGCCTCCATTCAT
NHX1-F	TGTGCTCTGGAGTGGTGGTA
NHX1-R	ATAGCCCCAATGGCTAGGTG
Actin-F	TTTGAGACTTTCAATGTGCCCGCC
Actin-R	TAGCATGTGGGAGTGCATAACCCT
